# RSM-Optimized Ultrasonic-Assisted Extraction for High-Efficiency Recovery of Pb, Cd, and As in Complex Systems

**DOI:** 10.1021/acsomega.6c03720

**Published:** 2026-06-09

**Authors:** Banpot Klinpratoom, Phitchan Sricharoen, Jintapat Nateewattana, Woravith Chansuvarn, Tanutta Amnuaywattanakul, Paisan Kanthang, Anchana Kuttiyawong, Titiya Meechai, Wongnapa Nakyai, Kanchana Suksri, Prawit Nuengmatcha, Nunticha Limchoowong

**Affiliations:** † Department of Chemisty, Faculty of Science, 37692 Srinakharinwirot University, Bangkok 10110, Thailand; ‡ Cosmetic and Herbal Innovation Center, Division of Health, Cosmetic and Anti-Aging Technology, Faculty of Science and Technology, 187381 Rajamangala University of Technology Phra Nakhon, Bangkok 10800, Thailand; § Faculty of Science and Technology, 187381 Rajamangala University of Technology Phra Nakhon, Bangkok 10800, Thailand; ∥ Faculty of Dentistry, 232369 Bangkokthonburi University, Thawi Watthana, Bangkok 10170, Thailand; ⊥ Faculty of Integrative Medicine, 65171 Rajamangala University of Technology Thanyaburi, Pathum Thani 12130, Thailand; # Division of Pharmacology and Biopharmaceutical Sciences, Faculty of Pharmaceutical Sciences, 37688 Burapha University, Chonburi 20131, Thailand; ¶ Center of Excellence in Nanomaterials Chemistry, Faculty of Science and Technology, 187416 Nakhon Si Thammarat Rajabhat University, Nakhon Si Thammarat 80280, Thailand

## Abstract

Toxic trace metals such as lead (Pb), cadmium (Cd), and arsenic (As) represent persistent environmental contaminants associated with significant human health risks. Reliable and rapid analytical strategies are therefore essential for toxicological surveillance of consumer and environmental matrices. In this study, a green and process-intensified ultrasonic extraction method was developed for the efficient recovery and determination of Pb, Cd, and As from complex matrices including herbal materials, cosmetic products, beverages, and wastewater, using Atomic Absorption Spectrometry. The approach utilizes ultrasonic cavitation to accelerate metal desorption and mass transfer, enabling rapid extraction under mild conditions with reduced reagent consumption. Galangal was selected as a representative plant matrix to establish and validate the analytical framework. A rotatable central composite design based on response surface methodology was applied to evaluate the influence of nitric acid concentration (0.40–1.20 M), ultrasonic time (5–15 min), and acid volume (20–40 mL) on metal recovery. The statistical model demonstrated strong predictive capability and identified acid concentration and solvent volume as significant factors influencing extraction performance (*p* < 0.05). Ultrasonic irradiation significantly enhanced metal release from the matrix through cavitation-induced disruption and accelerated diffusion processes. The optimized extraction conditions (0.68 M HNO_3_, 11 min ultrasonic time, and 32 mL acid volume) yielded predicted recoveries of 97.13%, 102.09%, and 103.26% for Pb, Cd, and As, respectively, with corresponding experimental recoveries of 95.17 ± 2.37%, 97.50 ± 5.13%, and 98.63 ± 0.25%. The developed method showed a very low matrix effect. The relative expanded uncertainty of metal extraction under optimized conditions ranged from 5.60 to 10.36%, which is within the acceptable criteria (≤15%). Based on the evaluation of trueness through comparison with results obtained from homogeneous proficiency testing (PT) materials, the developed method exhibited acceptable trueness for the quantitative determination of all trace metals, with *z*-scores less than 2. Application of the method to real samples demonstrated consistent analytical performance across seven galangal varieties and multiple complex matrices, with recoveries ranging from 87.00 to 109.00% (% RSD = 0.08–6.82). These findings demonstrate that ultrasonic processing can provide a rapid, low-reagent, and environmentally responsible extraction strategy for toxic trace metal determination. The developed protocol offers a practical analytical platform for toxicological monitoring, contaminant surveillance, and safety assessment of natural and industrial products.

## Graphical abstract

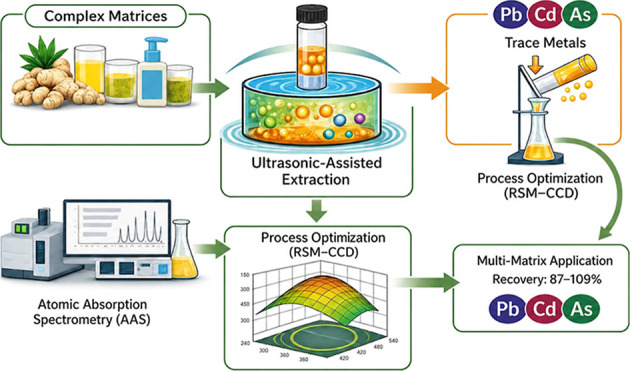

## 1 Introduction

The presence of toxic trace metals in environmental and consumer products has become an important public health concern due to their persistence, bioaccumulation potential, and well-documented toxicological effects. Elements such as lead (Pb), cadmium (Cd), and arsenic (As) are widely recognized as hazardous contaminants that can induce a broad range of adverse biological outcomes, including neurotoxicity, nephrotoxicity, carcinogenicity, and endocrine disruption following chronic exposure. Cd has also been shown to activate the innate immune system via the AIM2 inflammasome.^1^ These metals are not biodegradable and may accumulate in biological tissues over time, thereby increasing the risk of long-term toxicity, even at low concentrations. Consequently, reliable analytical strategies for monitoring toxic metal contamination in food, herbal medicines, cosmetics, and environmental matrices are essential for protecting human health and ensuring regulatory compliance.

Herbal materials and plant-derived products represent an important class of consumer commodities that may serve as potential exposure pathways for toxic metals. Medicinal plants are capable of absorbing and accumulating metal ions from soil and water through root uptake mechanisms, particularly in areas influenced by industrial activities, mining operations, agricultural inputs, and atmospheric deposition. As a result, the presence of toxic metals in botanical raw materials has attracted increasing attention from regulatory authorities and toxicologists. Contaminant monitoring in herbal matrices is particularly important because these products are often consumed directly or incorporated into dietary supplements, pharmaceuticals, and cosmetic formulations. Galangal (*Alpinia galanga* (L.)) is a widely used rhizomatous plant belonging to the Zingiberaceae family and serves as a representative example of botanicals frequently utilized in food, traditional medicine, and cosmetic preparations across Southeast Asia.^2^ The rhizome is valued for its characteristic flavor and its diverse bioactive constituents, including phenolic compounds, flavonoids, and essential oils, which have been associated with anti-inflammatory, antimicrobial, and antioxidant activities. Despite its beneficial properties, galangal—like many agricultural products—can accumulate environmental contaminants, including toxic metals, during cultivation.^3^ Several environmental sources such as contaminated irrigation water, industrial emissions, traffic-related pollution, and the use of agrochemicals have been identified as contributors to metal contamination in soil and crop systems.^4–6^ Therefore, the presence of trace levels of Pb, Cd, and As in herbal raw materials represents a potential safety concern that must be carefully monitored. To minimize health risks associated with toxic metal exposure, regulatory organizations have established maximum allowable limits for heavy metals in herbal materials. For example, the Thai Herbal Pharmacopoeia, consistent with recommendations from the World Health Organization, specifies permissible limits of 10 mg/kg for Pb, 0.3 mg/kg for Cd, and 4 mg/kg for As in galangal and related herbal products. Accurate determination of these contaminants requires efficient sample preparation techniques capable of isolating trace metals from complex plant matrices without introducing analytical bias or contamination.^7–9^

Sample preparation remains one of the most critical steps in trace-metal analysis. Conventional methods such as dry ashing,^10^ wet acid digestion,^11^ and microwave-assisted digestion^12,13^ have been widely employed to convert solid plant materials into solutions suitable for instrumental analysis. While these techniques provide effective matrix decomposition, they generally involve high temperatures, extended digestion times, and large volumes of concentrated acids. These conditions increase operational costs, raise safety concerns, and generate environmentally undesirable chemical waste.^14^ From both environmental and analytical perspectives, the development of alternative extraction approaches that reduce reagent consumption, shorten preparation time, and maintain high analytical accuracy is, therefore, highly desirable.

Ultrasonic-assisted extraction (UAE) has emerged as a promising green analytical technique for enhancing solid–liquid extraction processes.^15,16^ The technique relies on acoustic cavitation, where the formation, growth, and implosive collapse of microbubbles generate localized high temperatures, pressures, microjets, and shear forces in the liquid phase, thereby disrupting solid matrices and cell walls, improving solvent penetration, and significantly enhancing mass transfer for rapid metal release from solid matrices.^17^ As a result, UAE can accelerate analyte release from complex matrices under relatively mild conditions, enabling efficient extraction with lower acid concentrations and shorter processing times compared with traditional digestion methods. These characteristics make ultrasonic processing an attractive strategy for improving analytical sample preparation while supporting environmentally responsible laboratory practices.

Although ultrasonic extraction offers significant advantages, the performance of the technique strongly depends on multiple interacting operational parameters, including extraction time, solvent composition, and extraction volume. Systematic optimization of these parameters is therefore essential to ensure reliable analytical results. Response surface methodology (RSM) provides a robust statistical framework for modeling complex relationships between experimental variables and analytical responses. In particular, central composite design enables efficient evaluation of nonlinear interactions among process parameters with a relatively small number of experimental runs.^18–20^ When combined with desirability-based optimization, RSM can identify optimal operating conditions that simultaneously maximize extraction efficiency and analytical reliability. Although the UAE technique offers several advantages, there has been no study investigating the statistical optimization of UAE conditions using RSM for the extraction of toxic trace metals from complex sample matrices. Therefore, this study aimed to address this research gap.

Therefore, the objective of this study was to develop an environmentally responsible ultrasound-assisted extraction strategy for the efficient determination of toxic trace metals (Pb, Cd, and As) in complex matrices. Galangal was selected as a representative botanical matrix to establish and optimize the extraction model. The effects of key operating parameters—including nitric acid concentration, ultrasonic irradiation time, and solvent volume—were systematically optimized using response surface methodology with a central composite design. The resulting analytical protocol was subsequently evaluated for its extraction efficiency, reproducibility, and applicability to multiple sample systems, including herbal materials, beverages, cosmetic products, and wastewater samples. By integrating ultrasonic processing with statistical optimization, this work aims to provide a rapid and sustainable analytical approach for monitoring toxic metal contamination in consumer and environmental matrices.

## 2 Materials and Methods

### 2.1 Chemicals

Nitric acid (70%, AR grade) was procured from UNIVAR (Australia), while the standard solutions of Pb (1004 ± 3 mg/L), Cd (999 ± 4 mg/L), and As (999 ± 5 mg/L) were supplied by Inorganic Ventures (USA). All standards were traceable to NIST. Deionized (DI) water with a resistivity of 18.2 MΩ·cm was produced using a Milli-Q water purification system (Merck, Germany). The homogeneous proficiency testing (PT) materials representing food and herbal matrices were obtained from the Bureau of Quality and Safety of Food (BQSF), Department of Medical Sciences, Ministry of Public Health, Thailand, which serves as the ASEAN Food Reference Laboratory for heavy metals and trace elements. The materials had assigned values for the toxic trace metals Pb, Cd, and As.

### 2.2 Apparatus

An analytical balance (Model PA323C, OHAUS, Thailand), with a maximum capacity of 320 g and a readability of 0.001 g, was utilized for sample weighing. Ultrasonic-assisted extraction was conducted using an ultrasonic bath (Cavitator Ultrasonic Cleaner Model 5.5S, Mettler Electronics, USA) with a capacity of 20.8 L, operated at a frequency of 67 kHz and a maximum power output of 200 W. Quantitative determination of heavy metals was performed using an atomic absorption spectrometer (Model PinAAcle 900F, PerkinElmer, USA). All procedures were carried out under the operational parameters detailed in Table 1. The atomization, background correction, and zero-setting procedures were performed by introducing samples through a nebulizer into an air–acetylene flame, where the analytes were converted into free ground-state atoms for AAS measurement. Background correction was conducted using a deuterium (D_2_) lamp system integrated into the AAS instrument. Prior to analysis, the instrument was zeroed using a calibration blank to establish the baseline signal. During measurement, absorbance signals obtained from standards and samples were automatically corrected for background contribution by the instrument, and calibration was performed based on the blank-corrected absorbance values.

**Table 1:** AAS Conditions for Determination of Heavy Metals

parameters	heavy metals		
	Pb	Cd	As
wavelength (nm)	283.31	228.80	193.70
slit width (nm)	0.70	0.70	0.70
oxidant air flow (L/min)	10.00	10.00	10.00
acetylene flow (L/min)	2.50	2.50	3.30
calibration curve range (mg/L)	0.05–0.80	0.01–0.16	0.20–3.20

### 2.3 Raw Material

Fresh galangal rhizomes of the Kaset Daengyai variety, which were employed as the model for extraction optimization, were acquired from a local market, Thailand. The rhizomes were meticulously washed with distilled water, segmented into small pieces, and subsequently dried overnight in a hot-air oven maintained at 50 °C. The dried material was then pulverized into a fine powder and sieved to isolate particles of 150 μm (100 mesh) size. The resulting ground material was stored in a sealed container and preserved within a desiccator until further analytical procedures were conducted. Samples of beverages, cosmetics, and wastewater were randomly collected from locations in the vicinity of the university.

### 2.4 UAE Procedure

An accurately weighed amount of 0.5 g (±0.05 g) of dried galangal powder was transferred into a 100 mL Erlenmeyer flask and spiked with a multielement mixed standard solution containing 0.20 mg L^–1^ Pb, 0.04 mg L^–1^ Cd, and 0.80 mg L^–1^ As. The sample was then subjected to ultrasonic-assisted extraction in an ultrasonic bath at room temperature according to the experimental conditions generated from the central composite design (CCD), which consisted of three independent variables. After the extraction process was completed, the mixture was filtered through Whatman No. 1 filter paper (110 mm in diameter) and the resulting solution was analyzed for heavy metal content using atomic absorption spectrometry (AAS). The reagent blank was prepared using extraction solvents containing HNO_3_ at the corresponding concentrations, without the addition of the sample matrix. The blank solutions were then subjected to UAE under the conditions designed by the CCD. The nonspiked sample was prepared by mixing the powdered sample with diluted HNO_3_ solution at each concentration, without the addition of the metal standards, subsequently and analyzed under the same conditions as the reagent blank. The overall experimental workflow used for the extraction and analysis of Pb, Cd, and As in galangal samples is illustrated in Scheme 1. The concentrations of the target metals were evaluated in terms of percentage extraction recovery, calculated using eq 1.^21^

1 $$\%\;recovery=\frac{\left(C_{found}-C_{non‐spiked}\right)}{C_{spiked}}\times 100$$

where *C*_found_, *C*_nonspiked_, and *C*_spiked_ represent the metal concentration after the addition of the known standard in the real samples, the metal concentration in the nonspiked real sample, and the known concentration of the spiked standard, respectively.

**Scheme 1:**
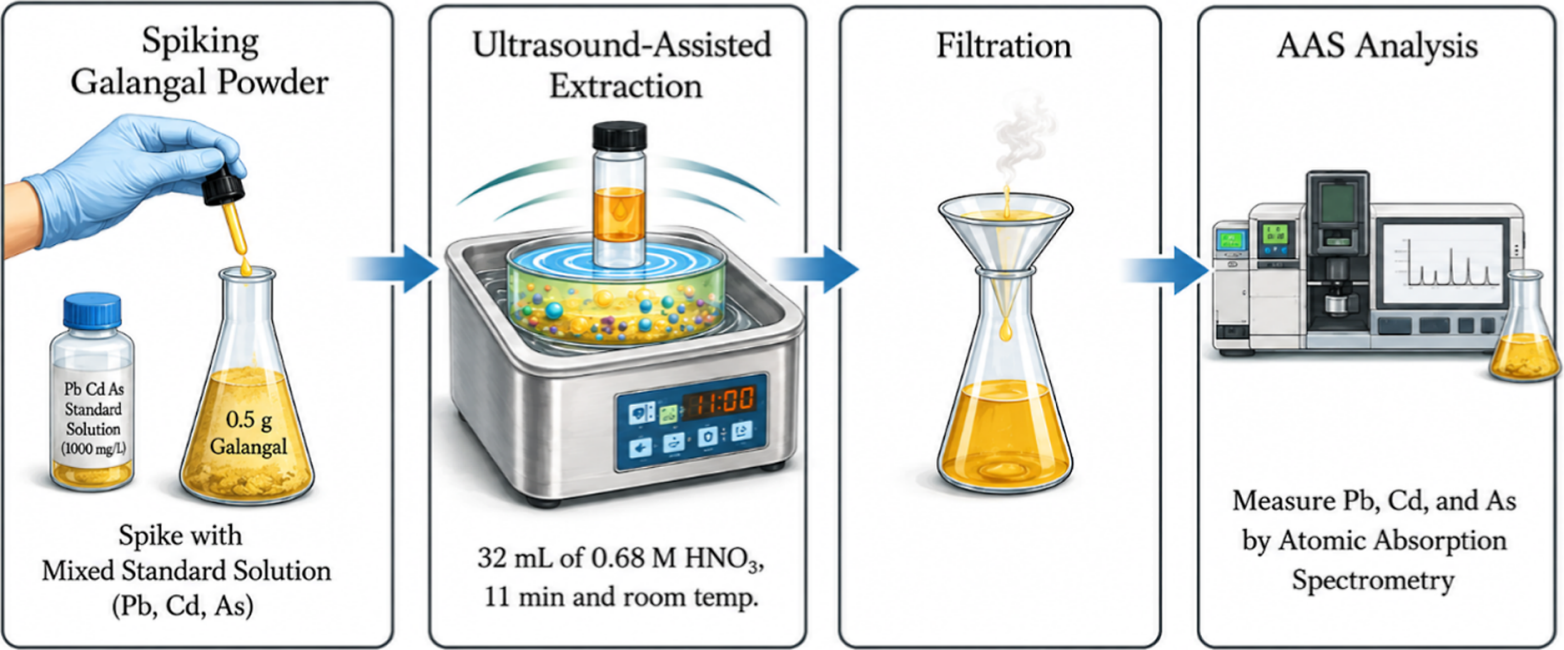
Schematic Representation of the Experimental Procedure for Ultrasonic-Assisted Extraction and Determination of Pb, Cd, and As in Galangal Samples Followed by Statistical Optimization and Analysis Using AAS. Photograph Courtesy of Banpot Klinpratoom. Copyright 2026

### 2.5 Statistical Experimental Design and Analysis

A rotatable central composite design (CCD) was utilized to structure the experimental framework, employing three independent variables: A, dilute HNO_3_ concentration (0.40–1.20 M); B, ultrasonic time (5–15 min); and C, acid volume (20–40 mL). This design aimed to optimize the conditions to achieve a high percentage of extraction recovery (% recovery) for subsequent analysis by Atomic Absorption Spectrometry. The total number of point experimental design could be calculated using the following equation

2 $$N=2^{k}+2k+C_{n}$$

where *N* is the total number of experiment point, *k* is the number of variables, and *C*_*n*_ is the number of repetitions of the experiments at the central point.

To optimize heavy metal extraction, a CCD model with three variables (*k* = 3) was employed, including eight replications at the factorial points (2^3^), six replications at the axial points (2(3)), and six replications at the central points (*C*_*n*_ = 6), resulting in a total of 20 runs of experiments (*N* = 2^3^ + 2(3) + 6 = 20). For statistical analysis, each of the variable was coded as five levels (-α, −1, 0, +1, + α). The coded of −1, 0, and +1 represents the lowest, central, and highest levels of the variables, respectively. The distance of each axial point (star point) from the center is α = (2^*k*^)^1/4^ (α = 1.68 with the number of variable (*k*) = 3)^21^. The experimental design coded levels and ranges of studied variables are shown in Table 2. A quadratic regression model equation (response surface model) for showing the influence of variable on the response and predicting the optimal point established was

3 $$Y=\beta_{0}+\beta_{1}A+\beta_{2}B+\beta_{3}C+\beta_{11}A^{2}+\beta_{22}B^{2}+\beta_{33}C^{2}+\beta_{12}AB+\beta_{13}AC+\beta_{23}BC$$

where *Y* is the predicted response (%extraction recovery), *A*, *B*, and *C* are the coded levels of the independent variables studied, β0 is the intercept coefficient, β1, β2, and β3 are linear coefficients, β11, β_22_, β_33_ are quadratic coefficients, β_12_, β_13_, β_23_ are cross-product coefficients.

**Table 2:** Three Independent Variables and Experimental Design Levels to Optimize UAE Conditions of Heavy Metals from Galangal^a^

variables	symbols	coded levels				
		-α	–1	0	+1	+α
dilute HNO_3_ concentration (M)	A	0.13	0.40	0.80	1.20	1.47
ultrasonic time (min)	B	2	5	10	15	18
acid volume (mL)	C	13	20	30	40	47

a α = 1.68.

### 2.6 Simultaneous Multiresponse Optimization of UAE Parameters Using RSM

Data results of all extraction experiments were obtained in triplicate, and the results are reported as mean values. The results obtained from CCD were used to analyze the response surface model. The goodness of fit of the model was evaluated by using the analysis of variance (ANOVA). Three-dimensional (3D) response surfaces and corresponding 2D contour plots were generated to illustrate the effect of the variables on the response (% recovery). The optimum conditions of the studied variables were determined by solving the response-surface model. An overlay contour plot and desirability function were applied to simultaneously optimize multiple responses under UAE conditions using Design-Expert version 13 software (State-Ease Inc., Minneapolis, MN, USA).

### 2.7 Predictive Model Validation

After extraction under the optimized conditions, the solution was quantitatively analyzed for the % recovery of Pb, Cd, and As (*n* = 3). The predicted responses and experimental (actual) values were compared for predictive model validation.

### 2.8 Method Validation

#### 2.8.1 Evaluation of Analytical Figures of Merit

A working standard solution of 0.05–0.80 mg/L Pb, 0.01–0.16 mg/L Cd, and 0.20–3.20 mg/L As was prepared by suitable dilution of intermediate mixed standard (Pb 50, Cd 10, and As 200 mg/L). Linearity of working ranges were assessed using OriginPro 2024 software (OriginLab Corporation, USA). The instrumental LOD and LOQ (mg/L) were calculated as 3 × SD/m and 10 × SD/m, respectively, where SD is the standard deviation of the lowest concentration of the metal and m is the slope of the calibration curve. These values were subsequently converted to an estimated sample basis (mg/kg) according to the sample mass and final extract volume, as shown in eq 4.

4 $$LOD,LOQ\;\left(mg/kg\right)=\frac{C\times V\times DF}{m}$$

where *C* denotes the instrumental LOD or LOQ value (mg/L), *V* is the final acid volume (mL), *m* is the sample mass (g), and *DF* is the postextraction dilution factor, if applicable.

Precision was expressed in terms of the % RSD of the slope of the calibration curve, which was evaluated in terms of repeatability (nine independent standard preparations, intraday precision) and reproducibility (analysis performed on different days, interday precision).

#### 2.8.2 Assessment of Matrix Effects

Matrix effects were assessed by comparing the slopes of calibration curves prepared in HNO_3_ solution (external calibration) with those of matrix-matched calibration curves. The matrix solution was prepared by extracting galangal powder without the addition of metal standard solutions under the optimum conditions obtained from RSM-CCD. After filtration, the extract was used to prepare matrix-matched calibration curves over the following concentration ranges: Pb (0.05–0.80 mg/L), Cd (0.01–0.16 mg/L), and As (0.20–3.20 mg/L). The percentage matrix effect was calculated according to eq 5.^22^

5 $$\%\;Matrix\;effect=\left[1-\left(\frac{Slope\;of\;external\;calibration}{Slope\;of\;matrix-matched}\right)\right]\times 100$$

#### 2.8.3 Measurement of Uncertainty

Measurement uncertainty was measured according to the procedures recommended in the EURACHEM/CITAC Guide.^23^ A cause-and-effect (Ishikawa fishbone) diagram illustrating the sources of uncertainty is listed in Figure 9. To estimate the combined uncertainty (Uc) of the analytical method, all major relative standard uncertainty components were calculated according to eq 6. These components were associated with the purity of the metal standard solution (Pstd), calibration linearity (Co), sample mass (Ms), micropipette volume (Px), volumetric flask volume (Vx), and glass pipet volume (Vf).

$$Uc=\sqrt{{\left(UPstd\right)}^{2}+{\left(UCo\right)}^{2}+{\left(U\;\mathrm{Ms}\right)}^{2}+{\left(UPx\right)}^{2}+{\left(UVx\right)}^{2}+(U_{Vf}{)}^{2}}$$ 6

**Figure 1:**
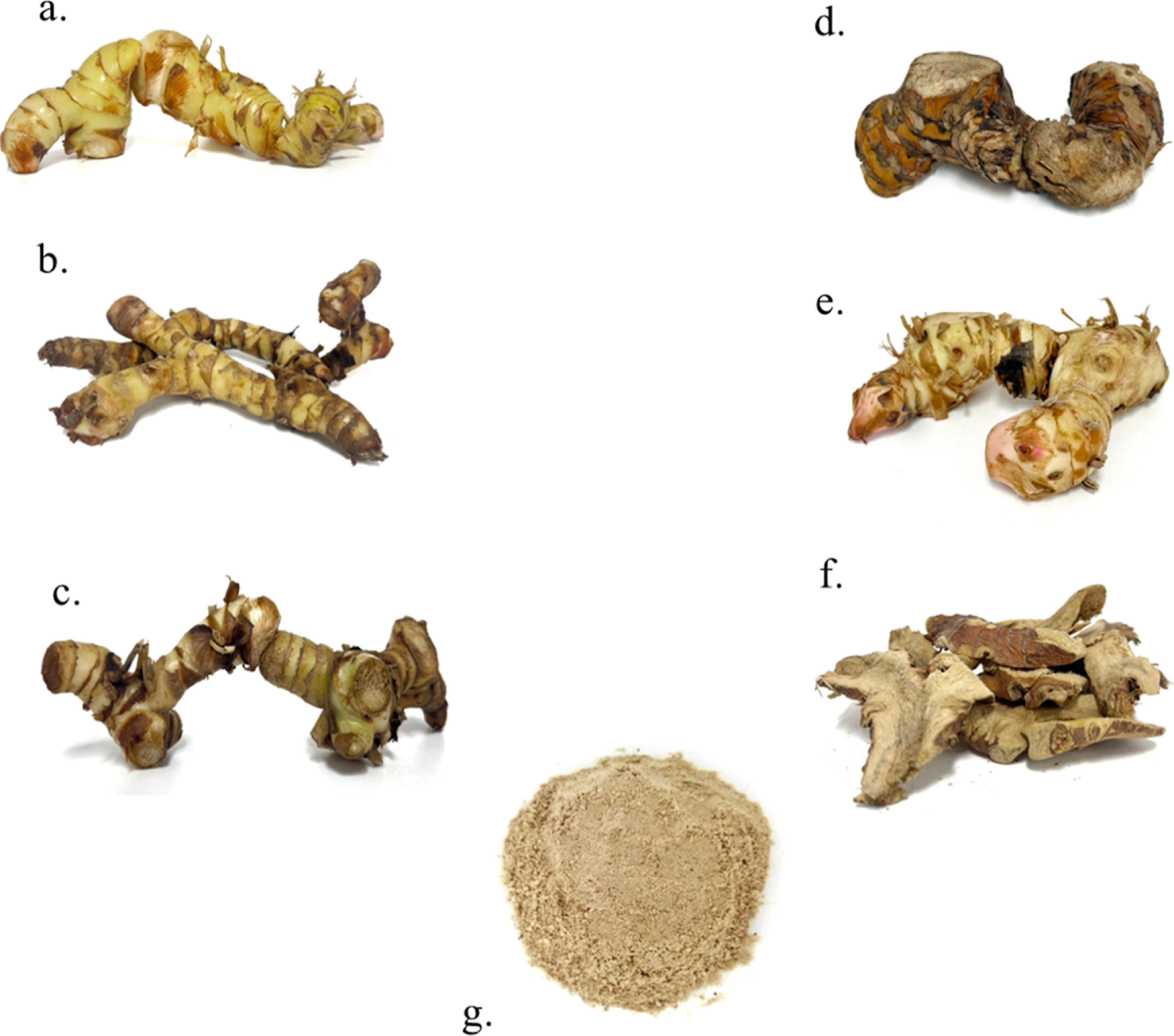
Morphology of seven galangal varieties used in real sample analysis. (a) Kha Leuang, (b) Kha Ling, (c) Kha Nahm, (d) Kha Tah Daeng, (e) Kha Kaset Daeng Yai, (f) Kha Tak Haeng (Dried galangal), and (g) Kha Phong (Dried galangal powder). Photograph courtesy of Banpot Klinpratoom. Copyright 2026.

**Figure 2:**
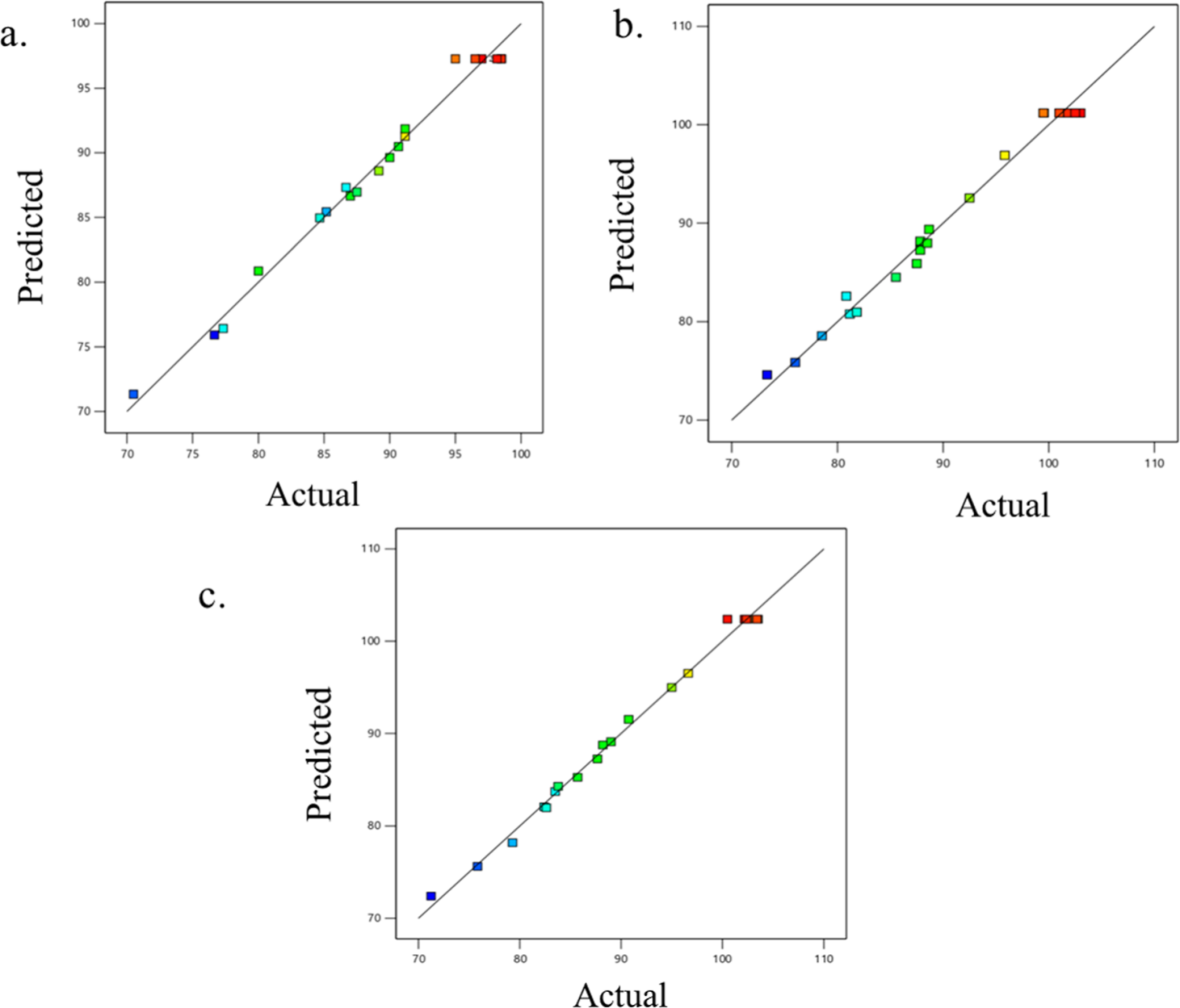
Relation between actual and predicted RSM values of %extraction recovery. (a) Pb, (b) Cd, and (c) As.

**Figure 3:**
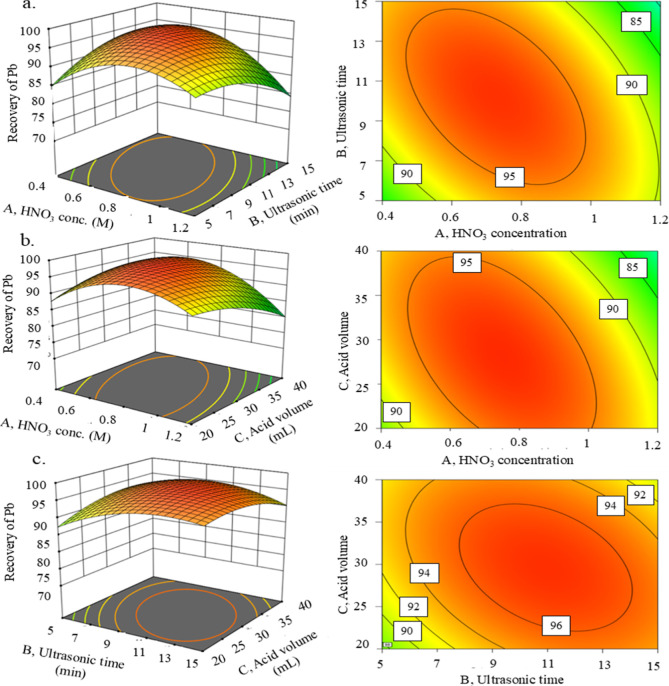
3D surface plots and corresponding contour plots showing the interaction effects of variables on the extraction recovery of Pb (a, b, c).

**Figure 4:**
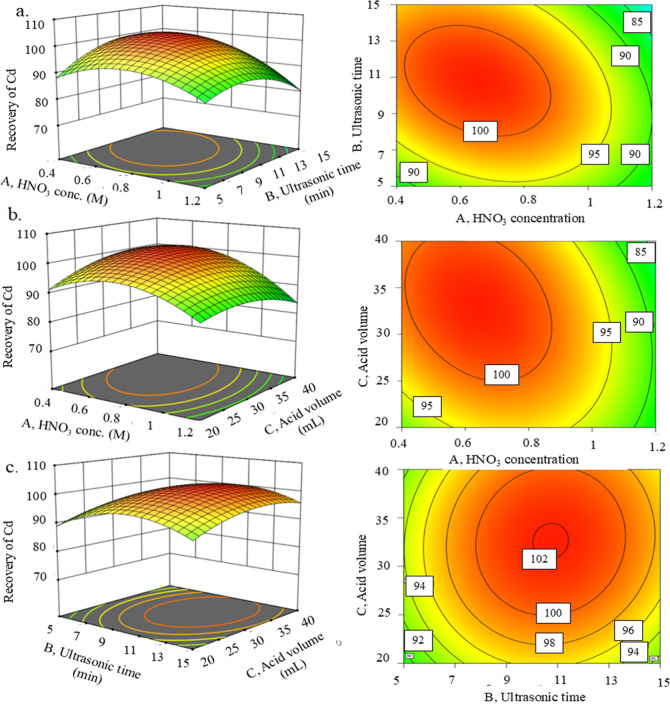
3D surface plots and corresponding contour plots showing the interaction effects of variables on the extraction recovery of Cd (a, b, c).

**Figure 5:**
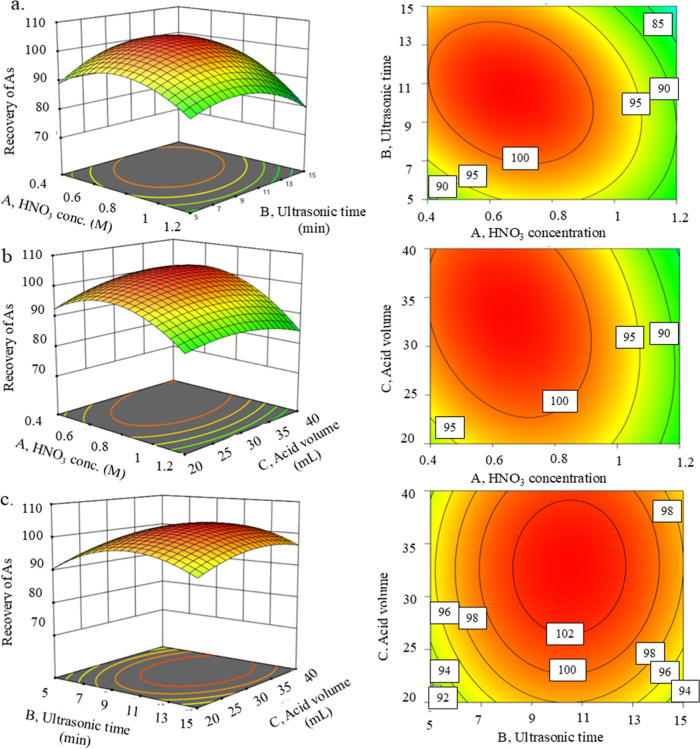
3D surface plots and corresponding contour plots showing the interaction effects of variables on the extraction recovery of As (a, b, c).

**Figure 6:**
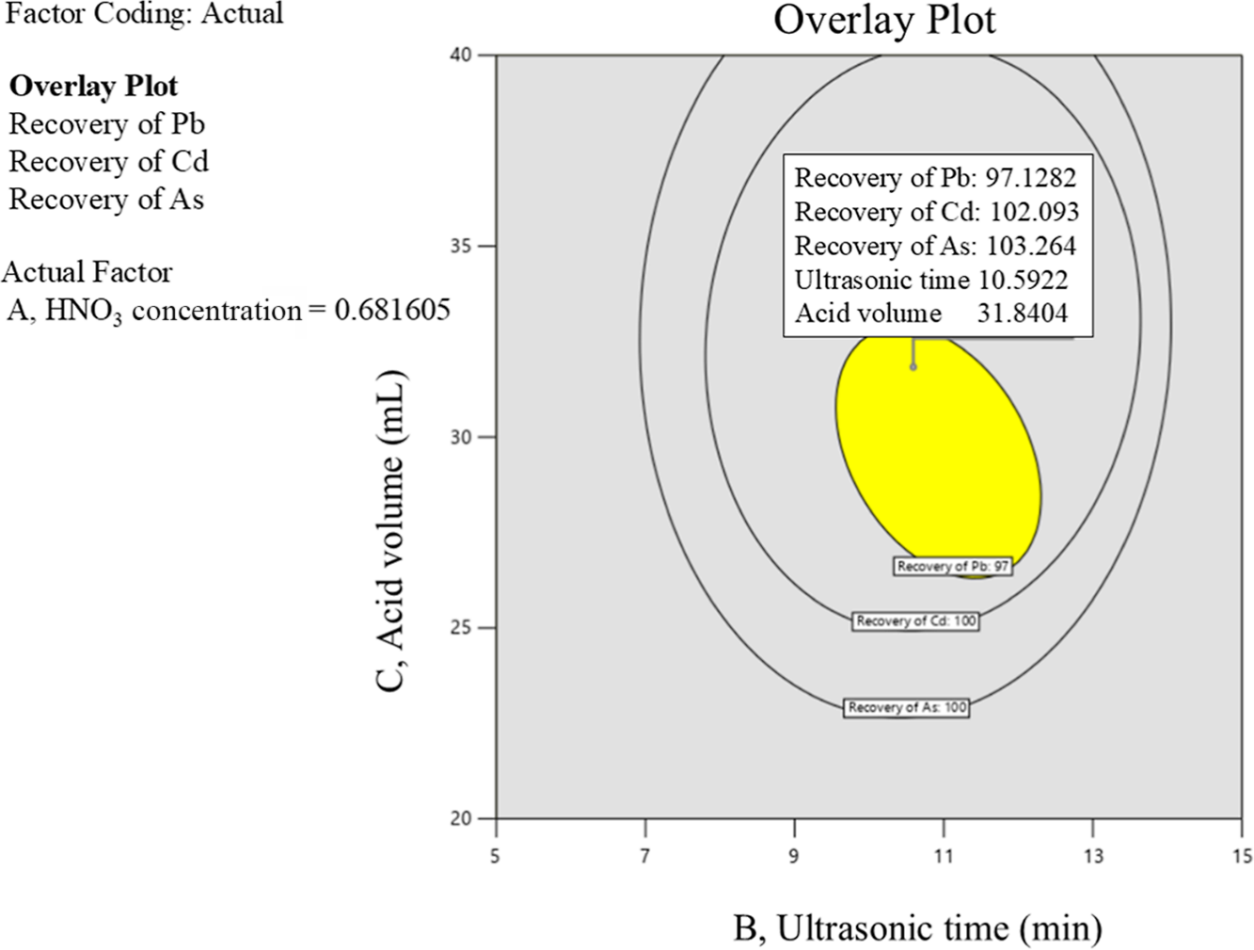
Optimum region obtained from overlay plots of multiple responses (Pb, Cd, and As) as a function of HNO_3_ concentration, ultrasonic time, and acid volume.

**Figure 7:**
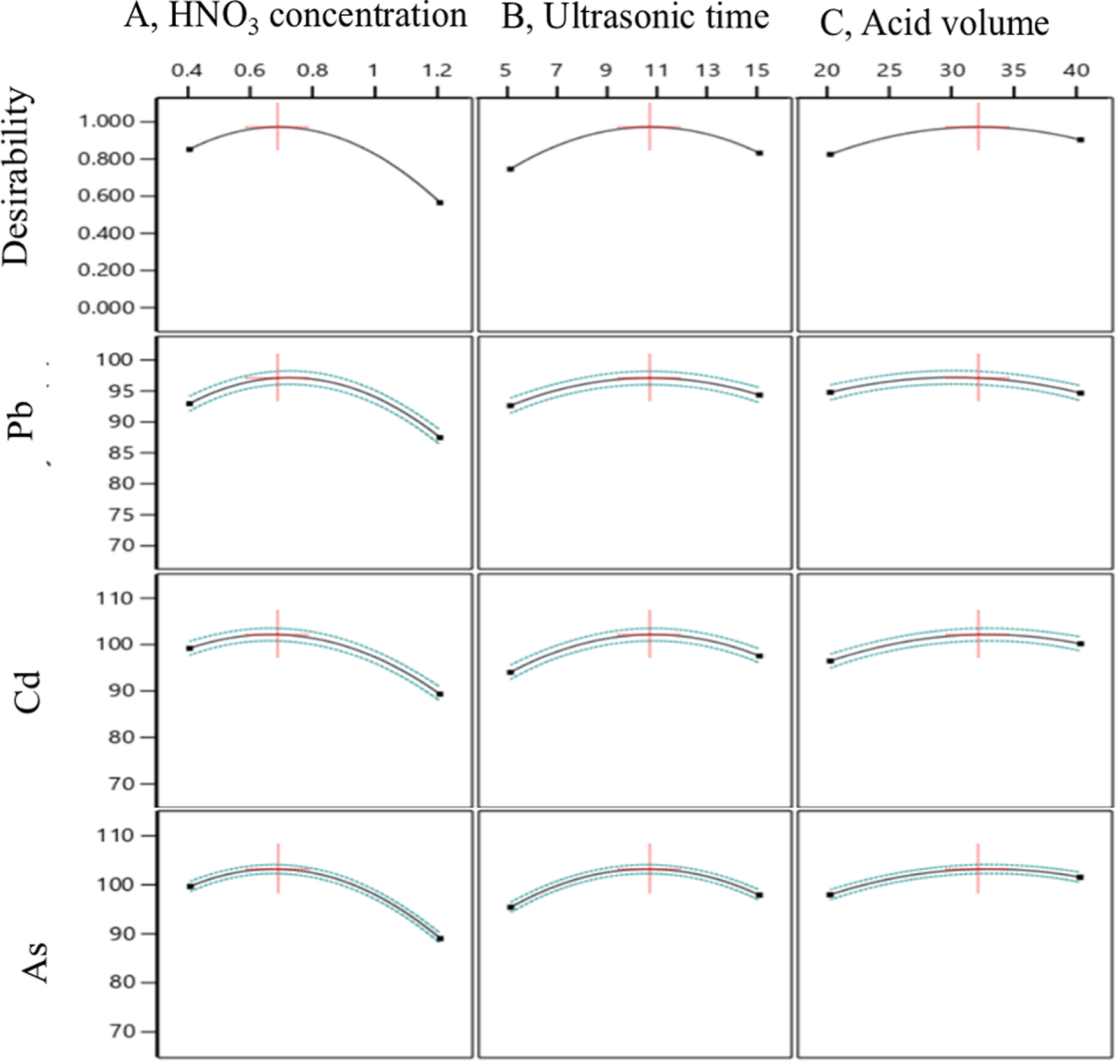
Desirability functions for multiresponse optimization of UAE conditions.

**Figure 8:**
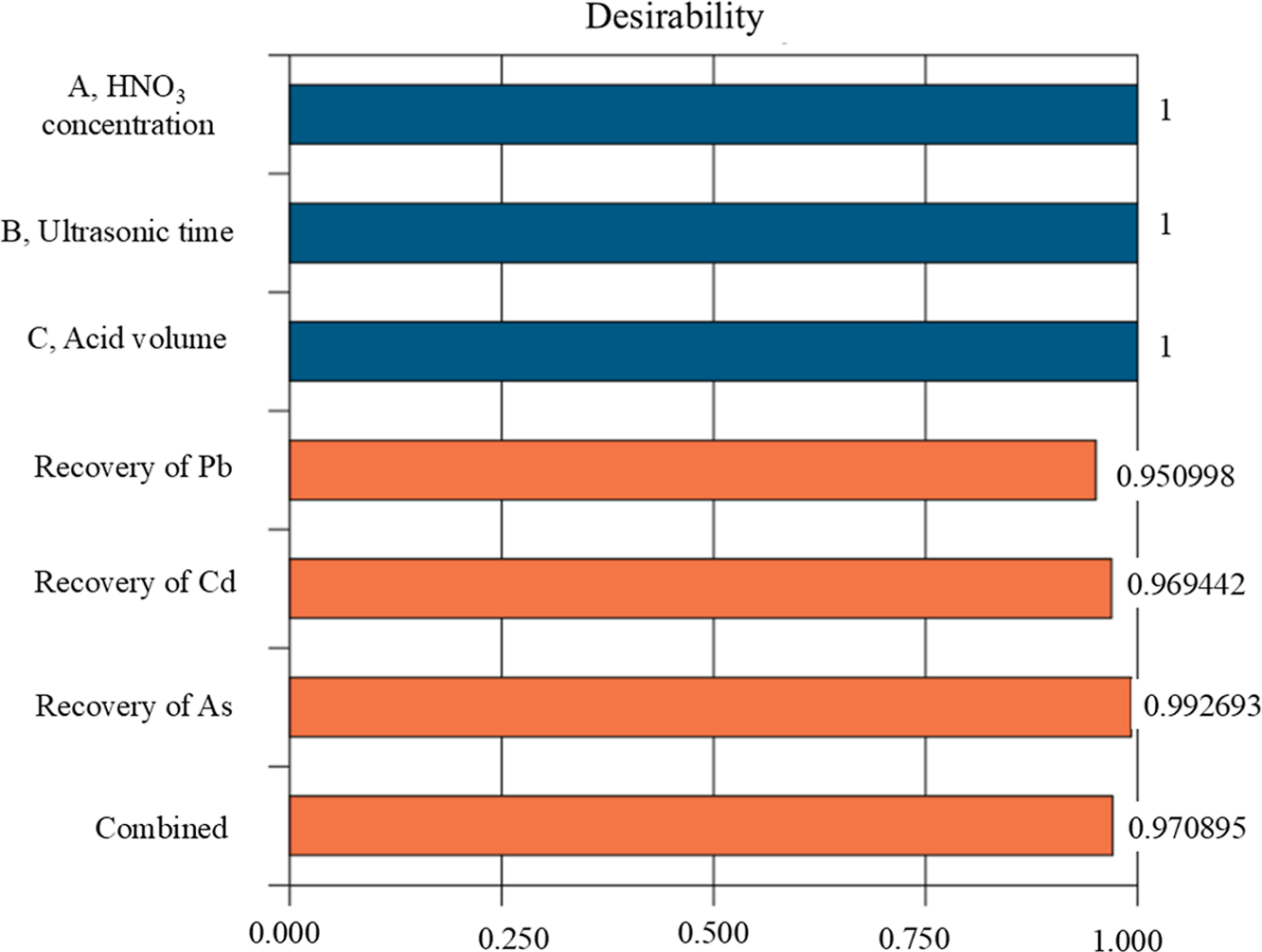
Pareto chart of the desirability functions of each variable for the responses.

**Figure 9:**
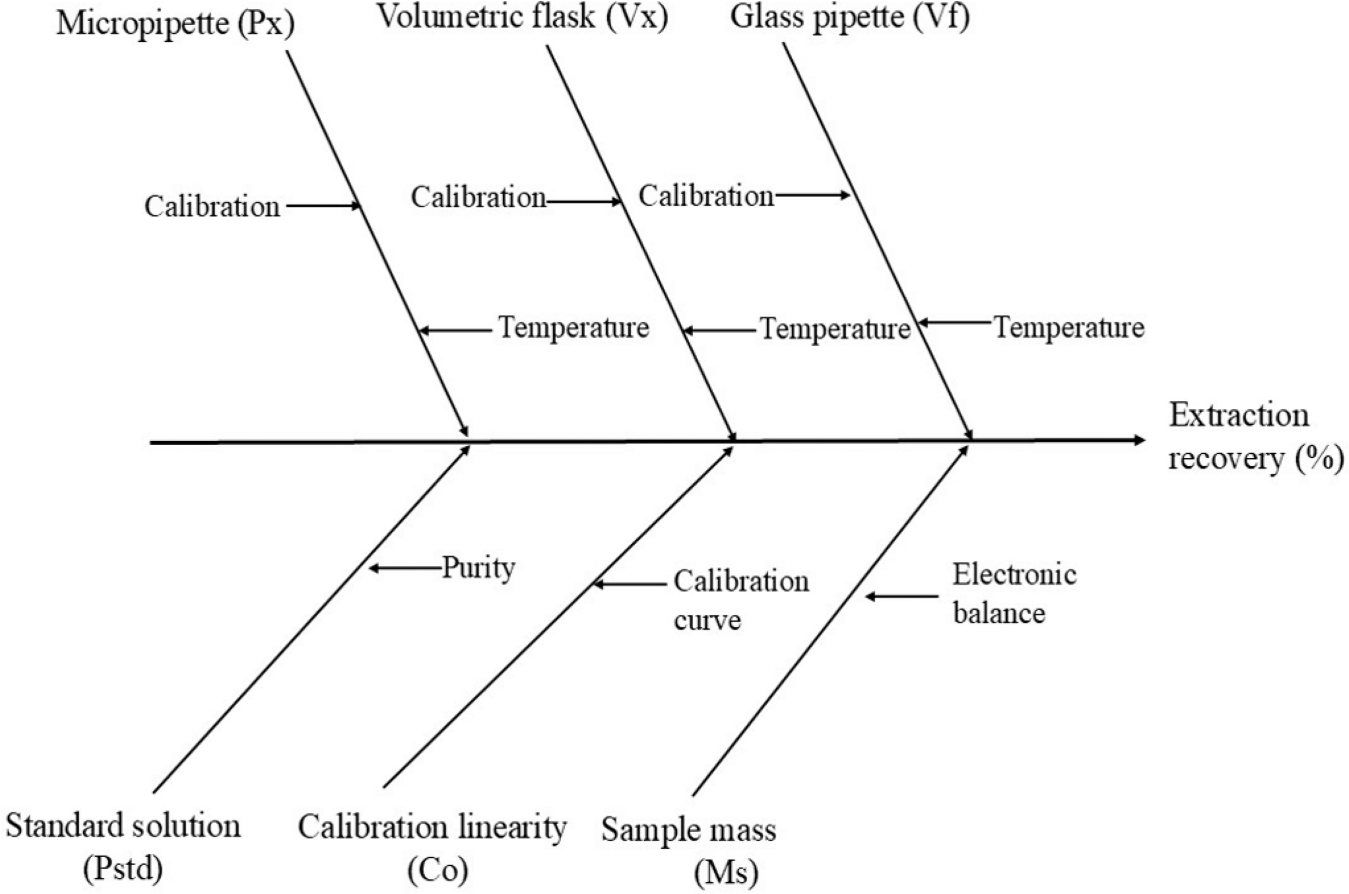
Ishikawa fishbone diagram illustrating the sources of measurement uncertainty in the analytical method.

The expanded uncertainty (Uex) at a 95% confidence level (coverage factor of *k* = 2) was calculated using eq 7.

7 Uex=Uc×2

The percentage extraction recoveries of Pb, Cd, and As under the RSM-optimized conditions were evaluated in terms of expanded uncertainty, and the analytical results were reported as follows.

The results are reported as results ± expanded uncertainty (Uex), and the percentage expanded uncertainty (%U) was calculated according to eq 8..

8 $$\%\;U=\frac{Uex}{C}\times 100$$

where Uex and *C* are the expanded uncertainty and the measured value of the measurand (% extraction recovery), respectively.

#### 2.8.4 Validation of the Trueness of the Proposed Method

Validation of trueness was performed by using PT materials representing food and herbal samples. The proposed UAE method was applied for sample preparation prior to trace metal determination, and the results were compared to those obtained using the reference microwave-assisted digestion (MAD) method.

For the reference method, the PT samples were digested by using concentrated HNO_3_ (5 mL) and H_2_O_2_ (1 mL) as the digestion solution. The digestion program consisted of four steps, as summarized in Table 10.

**Table 3:** CCD Experimental Design with Three Independent Variables and the Results Obtained from Extraction of Heavy Metals

	independent variables			responses (% extraction recovery)					
	A	B	C	Pb		Cd		As	
runs				actual	RSM	actual	RSM	actual	RSM
factorial points									
1	0.40 (−1)	15 (+1)	40 (+1)	91.17	91.28	95.83	96.89	96.62	96.51
2	1.20 (+1)	5 (−1)	20 (−1)	90.67	90.48	87.50	85.91	85.71	85.28
3	0.40 (−1)	5 (−1)	40 (+1)	87.00	86.65	87.83	87.27	88.21	88.76
4	1.20 (+1)	5 (−1)	40 (+1)	84.67	84.96	81.83	80.97	82.62	81.98
5	1.20 (+1)	15 (+1)	20 (−1)	85.17	85.43	78.53	78.56	79.29	78.20
6	0.40 (−1)	15 (+1)	20 (−1)	90.00	89.63	87.83	88.17	89.00	89.11
7	0.40 (−1)	5 (−1)	20 (−1)	77.33	76.42	81.17	80.78	82.42	82.08
8	1.20 (+1)	15 (+1)	40 (+1)	70.50	71.33	76.00	75.86	75.83	75.63
axial points									
9	0.80 (0)	10 (0)	13 (-α)	91.17	91.85	88.67	89.37	90.75	91.53
10	1.47 (+α)	10 (0)	30 (0)	76.67	75.91	73.33	74.60	71.25	72.40
11	0.13 (-α)	10 (0)	30 (0)	80.00	80.87	88.50	87.98	87.67	87.28
12	0.80 (0)	18 (+α)	30 (0)	87.50	86.96	85.53	84.51	83.79	84.31
13	0.80 (0)	10 (0)	47 (+α)	89.17	88.60	92.50	92.55	95.00	94.98
14	0.80 (0)	2 (-α)	30 (0)	86.67	87.32	80.83	82.60	83.50	83.74
central points									
15	0.80 (0)	10 (0)	30 (0)	97.00	97.27	103.00	101.21	102.17	102.38
16	0.80 (0)	10 (0)	30 (0)	96.50	97.27	101.00	101.21	103.40	102.38
17	0.80 (0)	10 (0)	30 (0)	98.17	97.27	102.50	101.21	100.50	102.38
18	0.80 (0)	10 (0)	30 (0)	98.50	97.27	101.83	101.21	102.35	102.38
19	0.80 (0)	10 (0)	30 (0)	95.00	97.27	99.53	101.21	103.50	102.38
20	0.80 (0)	10 (0)	30 (0)	98.50	97.27	99.50	101.21	102.50	102.38

**Table 4:** ANOVA and Regression Coefficients of the Response Surface Models for Extraction Recovery

factors	Pb_Re_		Cd_Re_		As_Re_	
	coefficients	*p*-value	coefficients	*p*-value	coefficients	*p*-value
intercept	97.27		101.21		102.38	
Linear						
A	–1.47	0.0011	–3.98	<0.0001	–4.42	<0.0001
B	–0.1051	0.7533	0.5681	0.1926	0.1669	0.5619
C	–0.9664	0.0140	0.9456	0.0424	1.03	0.0042
quadratic						
A^2^	–6.68	<0.0001	–7.04	<0.0001	–7.97	<0.0001
B^2^	–3.58	<0.0001	–6.24	<0.0001	–6.49	<0.0001
C^2^	–2.49	<0.0001	–3.62	<0.0001	–3.23	<0.0001
cross-product						
AB	–4.56	<0.0001	–3.68	<0.0001	–3.53	<0.0001
AC	–3.94	<0.0001	–2.86	0.0003	–2.50	<0.0001
BC	–2.15	0.0005	0.5584	0.3179	0.1819	0.6276
*p*-value (Model)	<0.0001		<0.0001		<0.0001	
*p*-value (lack of fit)	0.7691		0.4814		0.5958	
*R* ^2^	0.9878		0.9866		0.9944	
adjusted *R* ^2^	0.9769		0.9744		0.9893	
predicted *R* ^2^	0.9576		0.9380		0.9761	
C.V. (%)	1.36		1.68		1.14	
Adeq precision	30.5214		25.0373		41.2430	

**Table 5:** Actual vs. Predicted RSM Values of Extraction Recovery under Optimized Conditions

response variables (% extraction recovery)	RSM value	actual value	deviation error (%)
Pb	97.13	95.17 ± 2.37	2.06
Cd	102.09	97.50 ± 5.13	4.71
As	103.26	98.63 ± 0.25	4.69

**Table 6:** Analytical figures of Merit of the Proposed Method

analytical parameters	Pb	Cd	As
linear concentration range (mg/L)	0.05–0.80	0.01–0.16	0.20–3.20
*R* ^2^	0.9992	0.9998	0.9992
LOD (mg/L)	0.0118	0.0005	0.0156
(mg/kg)	0.7552	0.0320	0.9984
LOQ (mg/L)	0.0392	0.0018	0.0518
(mg/kg)	2.5088	0.1152	3.3152
intraday % RSD	2.13	3.52	2.85
interday % RSD	3.50	2.05	2.53

**Table 7:** Analytical Parameters Obtained from Matrix Effect Evaluation

metals	external standard curve		matrix-matched curve		
	*R* ^2^	slope	*R* ^2^	slope	matrix effect (%)
Pb	0.9992	0.0170	0.9985	0.0181	6.08
Cd	0.9998	0.3126	0.9957	0.3187	1.91
As	0.9992	0.0061	0.9969	0.0064	4.69

**Table 8:** Relative Standard Uncertainty Contributions from All Significant Sources

	metals		
(relative standard uncertainty)^2^	Pb	Cd	As
(U_Pstd_)^2^	2.23 × 10^–6^	4.01 × 10^–6^	6.26 × 10^–6^
(U_Co_)^2^	2.15 × 10^–3^	7.75 × 10^–4^	2.68 × 10^–3^
(U_Ms_)^2^	1.85 × 10^–8^	1.85 × 10^–8^	1.85 × 10^–8^
(U_Px_)^2^	2.62 × 10^–5^	2.62 × 10^–5^	2.62 × 10^–5^
(U_Vx_)^2^	1.70 × 10^–6^	1.70 × 10^–6^	1.70 × 10^–6^
(U_Vf_)^2^	8.88 × 10^–7^	8.88 × 10^–7^	8.88 × 10^–7^
combined uncertainty (Uc)	4.65 × 10^–2^	2.80 × 10^–2^	5.18 × 10^–2^
expanded uncertainty (Uex)	9.30 × 10^–2^	5.60 × 10^–2^	10.36 × 10^–2^

**Table 9:** Expanded Uncertainty and Percentage Expanded Uncertainty of Extraction Recovery Obtained under the Optimized Extraction Conditions

metals	% extraction recovery	Uex	% U
Pb	95.17	±8.85	9.30
Cd	97.50	±5.46	5.60
As	98.63	±10.22	10.36

**Table 10:** PT Sample Preparation Conditions for Microwave-Assisted Digestion (MAD)

digestion step	time (min)	temperature (° C)	pressure (bar)	power (W)
I	5 (ramp)	75	30	1800
II	15 (ramp)	230	90	1800
III	15 (hold)	230	90	1800
IV	30 (cooling)	50	–	–

After digestion, the solution was filtered through a Whatman No. 1 filter paper and made up to 25 mL with deionized water in a volumetric flask prior to the determination of Pb, Cd, and As.

The trueness of the proposed method was validated by comparing the results for the three target metals (mg/kg) with those obtained using the reference sample preparation method and with the assigned values of the PT materials. The agreement between the measured values and the assigned values was further evaluated using the *z*-score, calculated as

9 $$z=\frac{\left(x-Xi\right)}{\sigma }$$

where *x* is the measured value, Xi is the assigned value, and σ is the standard deviation for proficiency assessment.

According to commonly accepted criteria, |*z*|≤2 indicates satisfactory performance, 2<|*z*|<3 indicates questionable performance, and |*z*|≥3 indicates unsatisfactory performance.^24^

### 2.9 Analysis of Real Samples

To evaluate the performance of the proposed method for a specific sample type (matrix effect), the accuracy of the method was assessed by determining the percentage of extraction recovery of seven varieties of galangal. Their common Thai names are Kha Leuang, Kha Ling, Kha Nahm, Kha Tah Daeng, Kha Kaset Daeng Yai, Kha Tak Haeng (Dried galangal), and Kha Phong (Dried galangal powder). The morphology of seven varieties of galangal is shown in Figure 1. All galangal sample powders (0.50 ± 0.05 g) were spiked at three different concentrations (low, medium, and high levels standard solution, *n* = 3) of Pb (0.05, 0.20, 0.80 mg/L), Cd (0.01, 0.04, 0.16 mg/L), and As (0.20, 0.80, 3.20 mg/L). After extraction under the optimized conditions, the % recovery values were recorded to assess the accuracy of the proposed method. To apply the optimized UAE conditions to other type of real samples, the effect of sample matrices on the extraction process was examined. Three type of real samples (0.50 g), including food (herbal beverage), cosmetic product (body lotion), and wastewater (from the Saen Sab Canal, Bangkok, Thailand), were spiked with a known concentrations of heavy metals at low, medium, and high levels standard solution (*n* = 3). Subsequently the extraction process was performed under RSM-optimized conditions. The extraction efficiency of heavy metals in the three types sample matrices was expressed as percentage of extraction recovery (% ER). All measurements were performed in triplicate and the results were reported as the mean value with % RSD.

## 3 Results and Discussion

### 3.1 Model Fitting and Statistical Analysis

CCD for the experimental design and the experimental (actual) compared with predicted results (RSM) are shown in Table 3. As can be seen from the results, the actual extraction recovery of all responses agrees well with the RSM results for all experimental runs. The predicted response surface models for % recovery Pb, Cd, and As are illustrated in eqs 10–12). These model equations specify the relationship between %recovery and the UAE variables (including dilute HNO_3_ concentration, ultrasonic time, and acid volume).

10 $${Pb}_{Re}=97.27-1.47A-0.1051B-0.9664C-{6.68A}^{2}-{3.58B}^{2}-{2.49C}^{2}-4.56AB-3.94AC-2.15BC$$

11 $${Cd}_{Re}=101.21-3.98A+0.5681B+0.9456C-{7.04A}^{2}-{6.24B}^{2}-{3.62C}^{2}-3.68AB-2.86AC+0.5584BC$$

12 $${\mathrm{As}}_{Re}=102.38-4.42A+0.1669B+1.03C-{7.97A}^{2}-{6.49B}^{2}-{3.23C}^{2}-3.53AB-2.50AC+0.1819BC$$

where *A*, *B*, *C* are the coded units of dilute HNO_3_ concentration, ultrasonic time, and acid volume, respectively.

The statistical significance of the predictive model was assessed using the probability values (*p*-values) for the analysis of variance (ANOVA), as shown in Table 4. The *p*-values of the model were less than 0.0001, revealing a significant model fit.^25^ The goodness of fit of the response surface model was evaluated by determining the adjusted *R* ^2^ and predicted *R* ^2^ coefficients. The adjusted *R* ^2^ values for the Pb, Cd, and As extraction recoveries were 0.9769, 0.9744, and 0.9893, respectively. Adjusted *R* ^2^ coefficients greater than 0.90 demonstrate the goodness of fit of the model.^26^ In addition, the adjusted *R* ^2^ is in reasonable agreement with the predicted R^2^, with a difference of less than 0.20, demonstrating the strong model and its accuracy in predicting the response.^27^

Figure 2 shows the predicted extraction recovery from the model versus actual results. As can be seen in the scatter diagram, the predicted responses and the actual results are distributed randomly along and near a straight line, indicating good agreement between the two results within the range of the operating variables.^28^ The coefficient of variation (CV) directly influences the degree of precision in comparing the experiments and is a good index for the reliability of the experiments. A lower CV value indicates a greater reliability of the experiment. Relatively low CV values of all responses were recorded in the range from 1.14 to 1.68%, clearly indicating a better precision and greater reliability of the experiments.^29^ Adeq Precision was used to measure the signal-to-signal (response-to-deviation) ratio. A ratio greater than 4 is desirable.^29^ The ratios of 30.5214, 25.0373, and 41.2430 for Pb, Cd, and As, respectively, indicate that the model has an adequate signal and reliability for prediction. Additionally, the error analysis results show that the lack of fit was not significant (*p*-values >0.05), indicating that the model adequately predicts all responses^30^ (% Recovery of Pb, Cd, and As) within the Experimental Variables.

ANOVA showed that the linear terms (A, C, all except B of all responses), quadratic terms (A^2^, B^2^, and C^2^), and cross-product terms (AB, AC, all except BC of Cd and As) were significant model terms (*p* < 0.05). This statistical analysis indicates that the dilute HNO_3_ concentration and acid volume have a direct effect on the % extraction recovery.

### 3.2 Effect of UAE Conditions on the Extraction Recovery of Heavy Metals

Figure 3a shows the 3D response surface and contour plots for the interaction effect of the HNO_3_ concentration and ultrasonic time on the extraction recovery (%) of Pb. The results demonstrate that the recovery increases from 85% to 95% when the acid concentration and ultrasonic time are increased. Then, the value gradually decreases from 95% to 85% with an increase in HNO_3_ concentration (from 1 to 1.20 M).

Figure 3b,c show the effect of the combined effect between the acid volume and another variable on the extraction recovery. The 3D response and contour plots reveal that the recovery continuously increases as the acid volume increases. Then, the value gradually decreases with an increase in the acid volume (from 35 to 40 mL).

Figure 4a represents the 3D response surface and corresponding contour plots for the interaction effect of the HNO_3_ concentration and ultrasonic time on the extraction recovery (%) of Cd. The results demonstrate that the recovery increases from 90% to 100% when the acid concentration and ultrasonic time are increased. Then, the value gradually decreases from 100% to 85% with an increase in HNO_3_ concentration (from 0.85 to 1.20 M).

Figure 4b,c represent the effect of the combined effect between the acid volume and another variable on the extraction recovery. The 3D response and contour plots reveal that the maximum recovery (100%) was achieved when the acid volume ranged from 25 to 40 mL.

Figure 5a illustrates the 3D response surface and 2D contour plots for the interaction effects of HNO_3_ concentration and ultrasonic time on the extraction recovery (%) of As. The results demonstrate that the recovery increases from 90% to 100% when the acid concentration and ultrasonic time are increased. Then, the value gradually decreases from 100% to 85% with an increase in HNO_3_ concentration (from 0.85 to 1.20 M).

Figure 5b,c illustrate the effect of the combined effect between the acid volume and another variable on the extraction recovery. The 3D response and contour plots reveal that the maximum recovery (100%) was achieved when the acid volume ranged from 23 to 40 mL.

All results were consistent with those previously reported by Chemat and co-workers. (2017)^31^ and Radaelli et al. (2019),^32^ The decreasing trend in the extraction recovery at excessively high acid concentration, long ultrasonic time, and large acid volume is due to the increased dissolution of organic constituents from the herbs, such as phenolic compounds, humic substances, sugars, and other complex organic compounds. These compounds can interact with heavy metal ions to form complexes or generate colloids, which are present in forms that are difficult to accurately quantify by AAS.^31,32^

### 3.3 Multiresponses Optimization of UAE Conditions by RSM

The overlay plot and desirability function approaches were applied to identify the optimal extraction parameters for multiple variables and responses, integrating all goals combined into a single condition. Numerical optimization identified the optimal point at which the desirability function is maximized.^31^

Figure 6 shows the overlay contour plot of multiple responses for the optimal extraction conditions. The intersection region (yellow area) in the overlay plot represents the region of optimal conditions for the three variables (dilute HNO_3_ concentration, acid volume, and ultrasonic time) and three responses (% extraction recovery of Pb, Cd, and As). Based on the overlay plot, the optimal UAE conditions for achieving high extraction recovery of Pb, Cd, and As were determined to be 0.68 M acid concentration, 32 mL acid volume, and 11 min ultrasonic time. The predicted maximum % recovery of heavy metals was recorded as 97.13, 102.09, and 103.26, respectively. The results indicated that the optimal UAE conditions for toxic metal extraction determined by RSM-CCD required only a low concentration of dilute acid (0.68 M HNO_3_), which is easier to handle. In addition, the technique requires a short extraction time (11 min) and can be performed at room temperature, thereby reducing the evolution of environmentally harmful gases (carbon dioxide and nitrogen oxides) associated with concentrated acid–matrix reactions and elevated temperature.^14^ Therefore, the proposed technique can be considered an environmentally friendly alternative method.

The accuracy of the predicted optimal conditions was assessed using the desirability values, with values closer to 1, confirming the accuracy of the optimization.^33^ Figure 7 shows the desirability function curves under optimal conditions (red plus sign), as identified from the overlay plot, which were closer to 1 for three responses. Figure 8 illustrates the Pareto chart showing the desirability of each variable for the responses. All three responses combined into a single desirability function (combined desirability = 0.971), where a value closer to 1 indicates high accuracy of optimization.^32^

### 3.4 Model Validation

After extraction under optimized conditions, the results showed excellent agreement between the predicted RSM and actual values (% deviation error <5%), confirming the validity and adequacy of the model (Table 5).

### 3.5 Method Validation

#### 3.5.1 Analytical Figures of Merit

Table 6 shows the analytical figures of merit for the proposed method. The results demonstrated its high sensitivity and precision. Furthermore, the LOD and LOQ values covered the maximum permissible limit of heavy metal contamination in herbal medicines, as defined by the WHO guidelines and Thai Herbal Pharmacopoeia (Pb < 10 mg/kg, Cd < 0.3 mg/kg, and As < 4 mg/kg).^8,9^

#### 3.5.2 Matrix Effect

Table 7 presents the coefficient of determination (*R* ^2^), slope, and percentage matrix effect values obtained from the external standard calibration curves and the matrix-matched calibration curves. The results showed good agreement between the *R* ^2^ and slope values of the two calibration approaches. In addition, the method exhibited low matrix effect percentages, indicating negligible matrix interference in the determination of target metals.

#### 3.5.3 Measurement Uncertainty

The relative uncertainty of the linearity of the calibration curve (Uco)^2^ was the major contributor to the expanded uncertainty (Table 8). For multielement spiked samples prepared in the same batch, the relative standard uncertainties associated with sample mass (Ms), micropipette volume (Px), volumetric flask volume (Vx), and glass pipet volume (Vf) were identical for all analytes, whereas the combined uncertainty differed due to element-specific factors, including the purity of the stock standard solutions and calibration performance.

Table 9 presents the expanded uncertainty and relative expanded uncertainty of percentage extraction recovery for Pb, Cd, and As under the optimized conditions obtained by RSM. The relative expanded uncertainty values (%U) did not exceed ±15% of the measurands, demonstrating that the proposed method provides acceptable analytical precision, measurement reliability, and fitness for purpose under the optimized extraction conditions.^23^

#### 3.5.4 Trueness Validation of the Proposed Method

The trueness of the proposed method was validated by comparing the results for the three target metals to those obtained using microwave-assisted digestion (MAD) as the reference sample preparation method.

Table 11 presents the assigned values and measured metal contents obtained using the MAD and proposed UAE methods. The results demonstrated that no differences were observed between the values obtained by MAD and those obtained by the developed UAE method. In addition, the z-scores for the AAS results after UAE were lower than 2, indicating satisfactory agreement with the assigned values of the PT materials.^24^ Therefore, the developed method exhibited an acceptable trueness for the quantitative determination of trace metals.

**Table 11:** Comparison of Assigned and Measured Metal Values Obtained Using MAD and the Proposed UAE Methods

		content (mg/kg)			
^type of PT material^	^metals^	^assigned value (Xi)^	^MAD^	^UAE (x)^	*z*-score^a^
^food^	^Pb^	^0.64^	^1.23^	^0.79^	^1.38^
	^Cd^	^0.59^	^0.57^	^0.77^	^1.76^
	^As^	^13.2^	^12.07^	^12.54^	^–0.46^
^herbal^	^Pb^	^4.40^	^4.65^	^5.08^	^1.31^
	^Cd^	^0.50^	^0.40^	^0.51^	^0.12^
	^As^	^4.13^	^4.51^	^4.45^	^0.53^

a The z-scores were calculated by comparing the metal concentrations obtained after sample preparation using UAE to the assigned values of the PT materials. σ of the PT food matrix was 0.109 for Pb, 0.102 for Cd, and 1.43 for As. σ of the PT herbal matrix was 0.52 for Pb, 0.078 for Cd, and 0.60 for As.

#### 3.5.5 Analysis of Real Samples

The accuracy assessment by spiking heavy metals into seven varieties of galangal revealed satisfactory % extraction recovery in the range of 87.00 to 107.50% (Table 12). The results showed that high extraction recovery values within the acceptable range according to the AOAC standard criteria (80–110%).^34^ In addition, the % RSD ranged from 0.16 to 6.19%, which were within the Horwitz RSD (21.1%),^34^ confirming that the method effectively extracts and quantifies the analyte from the sample matrix. The extraction efficiency of heavy metals in three types of samples (beverage, body lotion, and wastewater) was evaluated to examine the applicability of the proposed method for the extraction of other types of samples. The results in Table 13 show that no detectable levels of metals were found in any samples (nonspiked samples). The % ER values of the spiked samples were in the ranges of 87.50–101.25 (% RSD 0.17–3.04), 89.40–109.00 (% RSD 0.16–2.18), and 91.00–107.00 (% RSD 0.08–6.82) for chrysanthemum tea (herbal beverage), body lotion, and wastewater, respectively. The % ER values were within the acceptable range (80–110%) with % RSD values lower than the Horwitz RSD (21.1%). Therefore, the proposed method is applicable to food, cosmetic products, and wastewater samples, yielding a high percentage of extraction recovery similar to those obtained from herbal samples. In summary, the overall experimental results indicate that the UAE-RSM technique was successfully applied to optimize the extraction conditions for the determination of heavy metal contamination (Pb, Cd, and As) in samples with high recovery. This green method enables the extraction of heavy metals using a low acid concentration, within a very short time, and at room temperature. The proposed method is environmentally friendly and can be further utilized for the quality analysis of heavy metals in food samples. Another advantage is that the desirability function approach based on RSM enables the simultaneous optimization of multiple responses into a single optimum condition, thereby reducing the number of required experiments.

**Table 12:** Analysis of Real Galangal Samples under Optimized Extraction Conditions (ND = Not Detectable, ^a^Dilute 5-fold)

	Pb				Cd				As			
sample	spiked	found	% ER	% RSD	spiked	found	% ER	% RSD	spiked	found	% ER	% RSD
	(mg/L)		(*n* = 3)	(*n* = 3)	(mg/L)	(mg/L)	(*n* = 3)	(*n* = 3)	(mg/L)	(mg/L)	(*n* = 3)	(*n* = 3)
Kha Leuang	-	ND	-	-	-	ND	-	-	-	ND	-	-
	0.05	0.0457	91.33	1.26	0.01	0.0103	103.00	5.59	0.20	0.191	95.50	1.09
	0.20	0.191	95.50	1.60	0.04	0.0383	95.75	1.51	0.80	0.750	93.75	0.34
	0.80^a^	0.812	101.50	0.36	0.16^a^	0.160	100.00	1.84	3.20^a^	3.160	98.75	0.42
Kha Ling	-	ND	-	-	-	ND	-	-	-	ND	-	-
	0.05	0.0497	99.33	3.08	0.01	0.0093	93.00	6.19	0.20	0.189	94.50	0.53
	0.20	0.207	103.50	1.70	0.04	0.0413	103.25	1.40	0.80	0.778	97.25	0.32
	0.80^a^	0.827	103.38	0.92	0.16^a^	0.155	96.86	3.69	3.20^a^	3.107	97.09	0.25
Kha Nham	-	ND	-	-	-	ND	-	-	-	ND	-	-
	0.05	0.0470	94.00	2.13	0.01	0.0107	107.00	5.41	0.20	0.200	100.00	1.04
	0.20	0.194	97.00	1.03	0.04	0.0393	98.25	1.47	0.80	0.793	99.12	0.33
	0.80^a^	0.807	100.88	0.72	0.16^a^	0.158	98.75	1.84	3.20^a^	3.150	98.44	0.42
Kha Tah Daeng	-	ND^a^	-	2.04	-	ND	-	-	-	ND	-	-
	0.05	0.0490	98.00	1.76	0.01	0.0103	103.00	5.59	0.20	0.194	97.00	0.79
	0.20	0.174	87.00	0.34	0.04	0.0393	98.25	1.47	0.80	0.796	99.50	0.40
	0.80^a^	0.853	106.62		0.16^a^	0.172	107.50	1.68	3.20^a^	3.172	99.12	0.24
Kha Kaset Daeng Yai	-	ND^a^	-	-	-	ND	-	-	-	ND	-	-
	0.05	0.0520	104.00	3.85	0.01	0.0093	93.00	6.19	0.20	0.201	100.50	1.03
	0.20	0.177	88.50	1.49	0.04	0.0380	95.00	2.63	0.80	0.789	98.62	0.25
	0.80^a^	0.825	103.12	0.61	0.16^a^	0.163	101.88	1.77	3.20^a^	3.180	99.38	0.16
Kha Tak Haeng	-	ND	-	-	-	ND	-	-	-	ND	-	-
	0.05	0.0460	92.00	2.17	0.01	0.0107	107.00	5.41	0.20	0.194	97.00	0.79
	0.20	0.183	91.50	1.57	0.04	0.0367	91.75	1.57	0.80	0.780	97.50	0.34
	0.80^a^	0.773	96.62	0.37	0.16^a^	0.142	88.75	4.17	3.20^a^	3.143	98.22	0.24
Kha Phong	-	ND	-	-	-	ND	-	-	-	ND	-	-
	0.05	0.0517	103.33	1.12	0.01	0.0103	103.00	5.59	0.20	0.198	99.00	1.82
	0.20	0.199	99.50	2.10	0.04	0.0367	91.75	4.17	0.80	0.763	95.38	0.20
	0.80^a^	0.813	101.62	0.94	0.16^a^	0.147	91.88	3.94	3.20^a^	3.130	97.81	0.32

**Table 13:** Analysis of Other Types of Samples under Optimized Conditions

sample	Pb				Cd				As			
	spiked	found	% ER	% RSD	spiked	found	% ER	% RSD	spiked	found	% ER	% RSD
	(mg/L)	(mg/L)	(*n* = 3)	(*n* = 3)	(mg/L)	(mg/L)	(*n* = 3)	(*n* = 3)	(mg/L)	(mg/L)	(*n* = 3)	(*n* = 3)
Chrysanthemum tea	-	ND	-	-	-	ND	-	-	-	ND	-	-
	0.05	0.0442	88.40	3.04	0.01	0.0099	99.00	2.55	0.20	0.190	95.00	0.52
	0.20	0.175	87.50	2.42	0.04	0.0375	93.75	2.67	0.80	0.794	99.25	0.17
	0.80^a^	0.769	96.12	0.48	0.16^a^	0.162	101.25	1.08	3.20^a^	3.21	100.31	0.21
body lotion	-	ND	-	-	-	ND	-	-	-	ND	-	-
	0.05	0.0447	89.40	2.02	0.01	0.0109	109.00	1.83	0.20	0.194	97.00	2.18
	0.20	0.183	91.50	1.27	0.04	0.0408	102.00	1.21	0.80	0.797	99.62	0.38
	0.80^a^	0.746	93.25	0.98	0.16^a^	0.161	100.62	0.56	3.20^a^	3.21	100.31	0.16
wastewater	-	ND	-	-	-	ND	-	-	-	ND	-	-
	0.05	0.0468	93.60	6.82	0.01	0.0107	107.00	4.40	0.20	0.196	98.00	1.16
	0.20	0.182	91.00	3.97	0.04	0.0375	93.75	2.18	0.80	0.801	100.12	0.08
	0.80^a^	0.772	96.50	0.87	0.16^a^	0.159	99.38	0.36	3.20^a^	3.21	100.31	0.19

### 3.6 Comparison with Previous Studies

The results of method validation and extraction recovery obtained in this study were compared to those reported in the literature, as summarized in Table 14. The results show that the LOD and LOQ values demonstrated that the proposed method was capable of determining heavy metal contamination in herbs at low concentrations, comparable to those reported in previous studies. The proposed method also exhibited high precision, as indicated by the low % RSD. In addition, this method enables the extraction of heavy metals using a low acid concentration, within a much shorter time, and at a lower temperature (operating at room temperature) compared with previous studies. Hence, this green method is environmentally friendly and can be further applied for the quality analysis of heavy metals in food samples.

**Table 14:** Comparison of the Sample Preparation Method and the Method Validation Results Obtained from This Work and the Previous Studies

					method validation					
sample	sample preparation method	time	instrumental analysis	elements	LOD(mg/L)	LOQ(mg/L)	intraday RSD (%)	interday RSD (%)	ER (%)	ref.
herbal origin in capsule	wet digestion solvent: acid mixture of 65% HNO_3_ and 37% HCl (1:3) (boiled over a water bath at 95 °C)	4–5 h	-GFFA (Pb, Cd)- HGAAS (As)	Pb	-	-	-	-	106.00	Uddin
				Cd	-	-	-	-	96.00	et al.^11^
				As	-	-	-	-	102.00	
Chinese materia medica	microwave digestion Solvent: concentrated HNO_3_ and H_2_O_2_ (digested at 205 °C)	50 min	ICP-MS	Pb	-	0.0002	5.48	-	82.60–89.70	Chien
				Cd	-	0.0004	0.43	-	91.70–97.20	et al.^35^
				As	-	0.0050	4.40	-	84.70–87.80	
herbal tea	microwave digestion solvent: concentrated HNO_3_ and H_2_O_2_ (digested at 225 °C)	30 min	ICP-MS	Pb	0.0013	0.0038	3.10	-	100.00	Klilic
				Cd	0.0005	0.0017	4.70	-	88.00	et al.^36^
				As	0.0010	0.0035	4.10	-	106.00	
Gingseng	microwave digestion Solvent: concentrated HNO_3_ and H_2_O_2_	30–40 min	ICP-MS	Pb	0.20	-	8.90	-	83.30	Wu et al.^37^
				Cd	0.01	-	3.80	-	103.00	
				As	0.05	-	4.20	-	96.70	
Sappan wood	ultrasound-assisted digestion Solvent: oxidant mixture (HNO_3_:H_2_O_2_ (2:1)) (temperature 60 °C)	30 min	Flame AAS	Pb	0.0170	0.0403	3.90	2.60	92.80	Siriangkhawut et al.^17^
				Cd	0.0006	0.0019	3.50	2.30	100.60	
				As	-	-	-	-	-	
Galangal,beverage, cosmetic and wastewater	ultrasound-assisted extraction	11 min	Flame AAS	Pb	0.0118	0.0392	2.13	3.50	87.00–104.00	this study
	solvent: 0.68 M HNO_3_			Cd	0.0005	0.0018	3.52	2.05	87.00–107.50	
	(room temperature)			As	0.0156	0.0518	2.85	2.53	93.75–100.50	

## 4 Conclusion

This study presents an ultrasound-assisted green extraction strategy for the efficient determination of toxic trace metals (Pb, Cd, and As) in complex matrices. Response surface methodology combined with a central composite design successfully identified the key extraction parameters influencing analytical performance, with the nitric acid concentration and solvent volume exerting significant effects on metal recovery. Under the optimized conditions (0.68 M HNO_3_, 11 min ultrasonic time, and 32 mL acid volume), high extraction efficiencies were achieved with an overall desirability value close to unity, demonstrating the predictive capability and robustness of the developed model. In addition, the developed method exhibited a very low matrix effect. The relative expanded uncertainty was within the acceptable criteria, and satisfactory trueness was achieved for the quantitative determination of trace metals. These findings indicate that the proposed method provides accurate and reliable measurements comparable to established reference methods such as microwave-assisted digestion. Application of the optimized protocol to real samples resulted in recoveries ranging from 87.00 to 107.50% across multiple galangal varieties. Additional validation using beverage, cosmetic, and wastewater matrices further confirmed the reliability and versatility of the method for complex sample systems. Ultrasonic cavitation facilitated rapid metal release from solid matrices through enhanced mass transfer, enabling efficient extraction under mild conditions with reduced acid consumption and a shorter processing time compared with conventional digestion-based approaches. Overall, the proposed analytical strategy provides a rapid, environmentally responsible, and reliable method for determining toxic trace metals in diverse matrices. The developed method can be applied to the analysis of various samples, particularly edible herbs, medicinal herbs, and cosmetic products containing herbal ingredients, to evaluate metal contamination levels against the standard limits established by the Thai Herbal Pharmacopoeia and the World Health Organization (WHO), thereby helping to protect consumers’ health. Furthermore, future studies may explore the application of this method to a broader range of contaminants and sample matrices as well as assess the scalability of the process for potential industrial applications.

## Abbreviations Used

MΩ.cm: Megaohm centimeter
kHz: Kilohertz
W: Watt
AAS: atomic absorption spectrometry
HNO_3_: nitric acid
M: molarity (moles per liter)
LOD: limit of detection
LOQ: limit of quantification
% RSD: percentage of relative standard deviation
% ER: percentage of extraction recovery
AOAC: Association of Official Analytical Chemists
